# Risk factors of chronic postoperative pain after total knee arthroplasty: a systematic review

**DOI:** 10.1186/s13018-024-04778-w

**Published:** 2024-05-29

**Authors:** Junfei Li, Tingyu Guan, Yue Zhai, Yuxia Zhang

**Affiliations:** 1 Fudan University, Shanghai, China; 2 Zhongshan Hospital, Shanghai, China

**Keywords:** Chronic pain, Pain, postoperative, Arthroplasty, replacement, knee, Risk factor

## Abstract

**Background:**

There is a lack of relevant studies to grade the evidence on the risk factors of chronic pain after total knee arthroplasty (TKA), and only quantitative methods are used for systematic evaluation. The review aimed to systematically identify risk factors of chronic postoperative pain following TKA and to evaluate the strength of the evidence underlying these correlations.

**Methods:**

PubMed, Web of Science, Cochrane Library, Embase, and CINAHL databases were searched from initiation to September 2023. Cohort studies, case-control studies, and cross-sectional studies involving patients undergoing total knee replacement were included. A semi-quantitative approach was used to grade the strength of the evidence-based on the number of investigations, the quality of the studies, and the consistency of the associations reported by the studies.

**Results:**

Thirty-two articles involving 18,792 patients were included in the final systematic review. Ten variables were found to be strongly associated with postoperative pain, including Age, body mass index (BMI), comorbidities condition, preoperative pain, chronic widespread pain, preoperative adverse health beliefs, preoperative sleep disorders, central sensitization, preoperative anxiety, and preoperative function. Sixteen factors were identified as inconclusive evidence.

**Conclusions:**

This systematic review clarifies which risk factors could be involved in future research on TKA pain management for surgeons and patients. It highlights those factors that have been controversial or weakly correlated, emphasizing the need for further high-quality studies to validate them. Most crucially, it can furnish clinicians with vital information regarding high-risk patients and their clinical attributes, thereby aiding in the development of preventive strategies to mitigate postoperative pain following TKA.

**Trial registration:**

This systematic review has been registered on the PROSPERO platform (CRD42023444097).

**Supplementary Information:**

The online version contains supplementary material available at 10.1186/s13018-024-04778-w.

## Introduction

Total knee arthroplasty (TKA) is the most common surgical intervention for patients with end-stage osteoarthritis [1].Despite a positive outcome for most patients, a sizeable portion of individuals experience significant pain following TKA [2]. Previous studies showed that the percentage of patients with unfavorable long-term pain outcomes ranged 10% ∼ to 34% following knee replacement [3]. The International Association for the Study of Pain (IASP) defines chronic postoperative pain (CPSP) as pain that persists for more than 3 months after surgery, excluding other causes (e.g., infection, surgical failure, recurrence of malignancy, etc.) [4]. In addition to disruption of daily activities brought on by the pain itself, adverse or chronic pain outcomes following joint replacement are of great concern to orthopedic surgeons and their patients. Chronic postoperative pain is also associated with deterioration in physical, functional, and mental domains, which implies significant personal, social, and healthcare costs with the rising prevalence of knee replacement surgeries [5].

Understanding the risk factors affecting chronic postsurgical pain can help increase the clinical staff’s understanding of the field, which can help clinicians make better decisions and help patients reduce the risk of developing chronic pain. Previous pain guidelines have only recommended perioperative interventions without doing an integration of risk factors [6]. Earlier systematic reviews that applied quantitative measures to identify predictors of persistent pain after TKA, without considering the grading of evidence, may result in limited quality outcome [7].

Therefore, this study will conduct a systematic review and critical appraisal of the risk factors affecting chronic pain after TKA, and use the Newcastle-Ottawa Scale (NOS) and the Agency for Healthcare Research and Quality (AHRQ) checklist to quality rate the level of evidence in the included literature.

## Methods

This article used the PRISMA (Preferred Reporting Items for Systematic Reviews and Meta-analyses) statement to guide implementation and reporting [8].

### Data sources and search strategy

Five databases were searched (PubMed, Web of Science, Cochrane Library, Embase, CINAHL) from the time of the foundation of the database to July 2023. All pertinent keyword variations were used, including both the Medical Subject Headings (Mesh) of various databases as well as the free-text versions of these terms. Reference lists of selected studies and reviews were searched to find additional publications on the subject. Detailed information about the search strategy is shown in Appendix 1.

### Study selection and eligibility criteria

Studies meeting the following criteria were included: (1) cohort studies or case-control or cross-sectional studies; (2) patients undergoing total knee arthroplasty who are aged above 18 years old; (3) the outcome was defined as postoperative pain following total knee arthroplasty and follow-up had to be at least three months; (4) outcomes were predicted using preoperative, intraoperative or postoperative conditions. If total hip arthroplasty (THA) and total knee arthroplasty (TKA) patients were both included in the study, only TKA data were extracted. The exclusion criteria were as follows: (1) publications written in languages other than English and Chinese, (2) studies with incomplete methodology and full text not available. In addition, given the large number of possible confounding variables, cohort studies that failed to use a multivariate approach to assess risk factors were excluded.

### Screening and data extraction

The titles and abstracts of all preliminary identified studies were screened by two investigators (JL and TG) independently following the selection criteria. Any differences of opinion were settled by consensus or discussion with a third independent reviewer. *If there were multiple publications available, the most recent data were taken.* To gather pertinent data, a predesigned electronic data extraction form was used. If there were multiple publications available, the most recent data were taken. The following information was extracted: participant characteristics, risk factors, pain outcome measures, follow-up period, and study design.

### Assessing the risk of bias

The risk of bias assessment was independently assessed by two authors (JL and TG) in each included study by using the Newcastle Ottawa Quality Assessment Scale (NOS) and the checklist recommended by the Agency for Healthcare Research and Quality (AHRQ) [9].

The Newcastle-Ottawa Scale (NOS) is an important tool that evaluates case-control and cohort studies. It is composed of three main sections, which include a total of eight items. These sections cover various aspects of the study, including the selection of the study population, comparability, and exposure/outcome evaluation. The NOS uses a semi-quantitative star system to rate the study’s quality, with a maximum score of nine stars. Studies were categorized as high quality (7–9 points), moderate quality (4–6 points), and low quality (0–3 points) [10]. To evaluate the quality of the cross-sectional studies, we utilized the checklist recommended by the Agency for Healthcare Research and Quality (AHRQ). The AHRQ Risk of Bias Evaluation Tool assesses the risk of bias in five domains, including selection bias, implementation bias, follow-up bias, detection bias, and reporting bias. If the answer was “no” or “unclear”, the score was 0. If the answer is “yes”, the score is 1. Articles are rated as low (0–3), moderate (4–7), or high quality (8–11) [11].

### Data synthesis and analysis

Semi-quantitative methods were used to summarize the strength of evidence supporting the association between risk factors and chronic postoperative pain. The best evidence synthesis included variables that were examined using a multivariate approach in at least two studies and demonstrated a statistically significant association. Three criteria were used to quantitatively evaluate the evidence of risk factors for chronic pain following total knee replacement: (1) the number of studies evaluating the variables; (2) the standard of the scores for each variable under assessment; (3) the consistency of the relationship between the factors and chronic postoperative pain. When 75% of the studies evaluating the variable reported the same direction of association, associations were deemed consistent [12]. Variables analyzed using multivariate methods that yielded no association were also taken into account. The level of evidence on risk factors for postoperative chronic pain was categorized into the following four categories: (1) strong: consistent findings were found in ≥ 2 high-quality articles; (2) moderate: with consistent results between 1 high-quality article and ≥ 1 moderate quality article or ≥ 3 moderate or low-quality articles; (3) inconclusive: When observed associations are inconsistent or assessed in 1 high-quality, < 3 moderate-quality studies or only in low-quality studies; (4) no association: no significant association was found in the high-quality multivariate analysis, or at least 3 high-quality studies found no association in the univariate analysis.

## Results

### Study identification

**Fig. 1:**
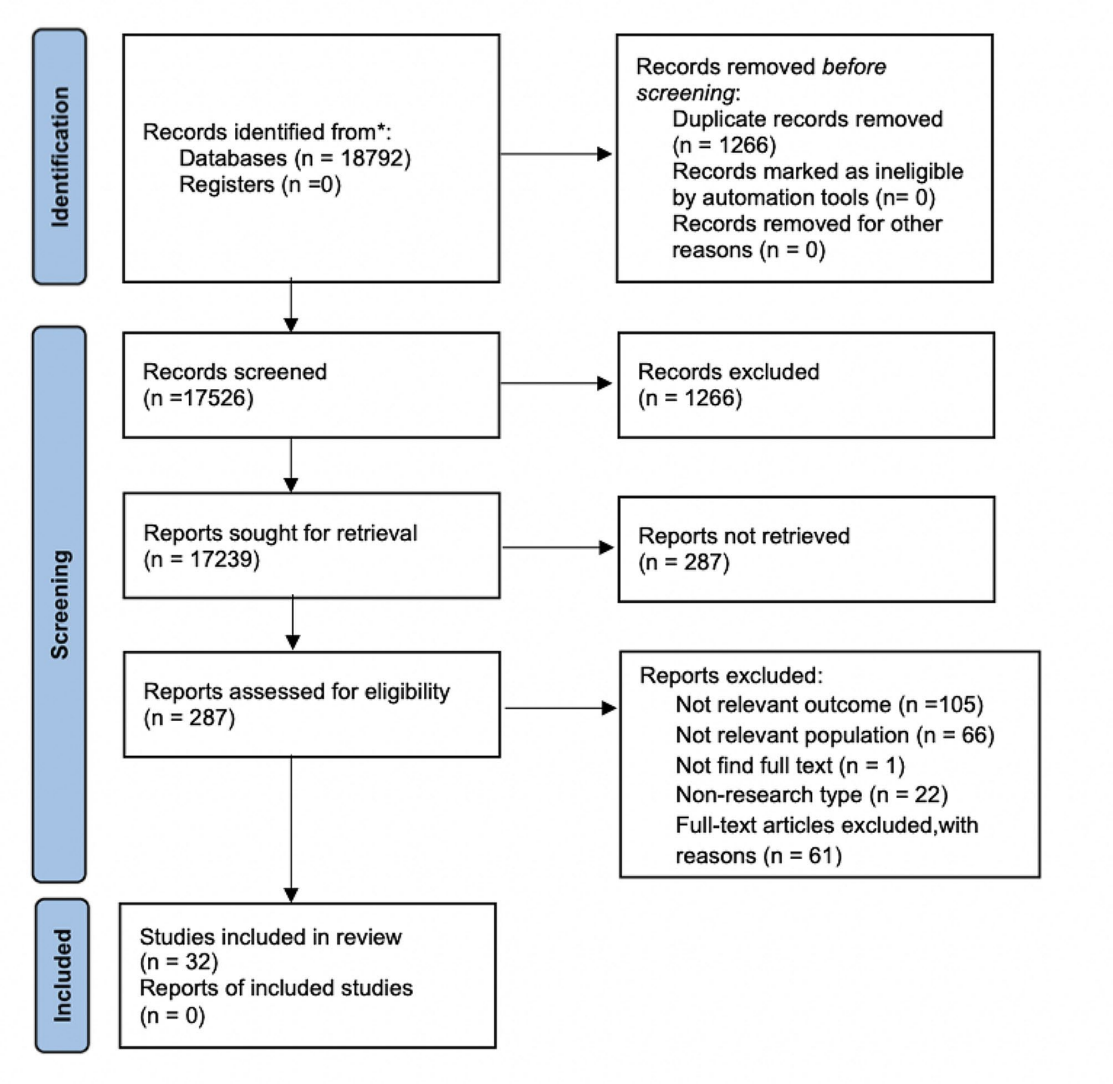
Flowchart of study selection

Database search returned 18,792 articles, and 7 relevant articles were obtained through supplements from other resources. A total of 17,526 articles were obtained after eliminating duplicates. 17,239 references were excluded from the initial screening by reading titles and abstracts, leaving 287 references for full-text review. Among the remaining articles, 105 did not cover the outcome of concern, 66 did not match the target population, the full text was not available for one study, and 61 were excluded for other reasons. Therefore, a total of 32 studies were included in the systematic evaluation including five cross-sectional studies, one case-control study, and 26 cohort studies. The flowchart and reasons for exclusion are delineated in Fig. 1.

### Study characteristics

A total of 32,645 patients who underwent primary total knee arthroplasty were enrolled in this study (see Table 1). The sample size ranged from 71 to 11,373. The commonly used outcome measurement instruments in the studies were the visual analog scale (VAS) (10 studies), Western Ontario and McMaster Osteoarthritis Index (WOMAC) pain scale (8 studies), and the Numerical Rating Scale (NRS) (7 studies). Five studies included total knee arthroplasty and total hip arthroplasty from which we extracted data for TKA. Study follow-up lasted a minimum of 3 months and a maximum of 10 years. Furthermore, 29 predictive factors associated with the development of postoperative chronic pain after TKA were identified.

**Table 1:** Characteristics of the 32 Literatures Included in the Systematic Review

No.	Study	Sample Size		Study Design	Outcome Measurement Instrument	Follow-up Duration	Quality Evaluation	Risk of Bias Score	Quality Assessment
1	Yan2023^32^		320	Prospective cohort study	NRS†	6 months	NOS ‡‡	7	High
2	Nishimoto2023^16^		71	Cohort study	KOOS‡	3 months	NOS ‡‡	9	High
3	Teimouri2023^29^		346	Case-control study	VAS*	6 months	NOS ‡‡	6	Moderate
4	Tang2023^28^		177	Prospective observational study	NRS†	6 months	AHRQ††	7	High
5	Chen2021^4^		220	Cohort study	VAS*	6 months	NOS ‡‡	9	High
6	Lindberg2021^13^		202	Longitudinal study	VAS*	4, 6 weeks, 3 months, 12 months	AHRQ††	8	High
7	Larsen2021^12^		131	Prospective cohort study	VAS*	12 months	NOS ‡‡	6	Moderate
8	Imai2021^9^		211	Prospective cohort study	VAS*	12 months	NOS ‡‡	4	Moderate
9	Also2021^1^		194	Prospective cohort study	VAS*	12 months	NOS ‡‡	9	High
10	Skrejborg2019^26^		352	Consecutive cohort study	NRS †	5 years	NOS ‡‡	9	High
11	Singh2019^23^		659	Prospective cohort study	KOOS‡	26 weeks	NOS ‡‡	9	High
12	Gungor2019^7^		578	Retrospective cohort study	NRS†	12 months	NOS ‡‡	5	Moderate
13	Buvanendran2019^3^		245	Prospective cohort study	NRS†	6 months	NOS ‡‡	9	High
14	Shim2018^22^		721	Prospective cohort study	OKS§	6 months	NOS ‡‡	7	High
15	Rice2018^20^		300	Prospective cohort study	WOMAC║	6, 12months	NOS ‡‡	9	High
16	Kornilov2018^11^		79	Longitudinal cohort study	NRS†	6 weeks, 3 months, 1 year	NOS ‡‡	9	High
17	Hofstede2018^8^		4183	Prospective cohort study	KOOS‡ VAS* WOMAC║ OKS§	12 months	NOS ‡‡	7	High
18	Jiang2017^10^		2080	Prospective cohort study	OKS§	1,5,10 years	NOS ‡‡	8	High
19	Dave2017^6^		241	Prospective cohort study	WOMAC║	12 months	NOS ‡‡	6	Moderate
20	Cremeans2016^5^		110	Prospective cohort study	WOMAC║	3 months	NOS‡‡	6	Moderate
21	Rajamäki2015^19^		134	Prospective cohort study	VAS*	1, 2 years	NOS‡‡	6	Moderate
22	Petersen2015^18^		78	Prospective cohort study	VAS*	12 months	NOS‡‡	7	High
23	Nashi2014^14^		357	Retrospective cohort study	KSS#	3 months, 6 months, 1 year and 2 years	NOS‡‡	9	High
24	Yakobov2014^31^		116	Prospective cohort study	WOMAC║	12 months	NOS‡‡	8	High
25	Noiseux2014^17^		193	Prospective cohort study	NRS†	6 months	NOS‡‡	8	High
26	Singh2013①^25^		11,373	Prospective cohort study	Mayo Knee question	2, 5years	NOS‡‡	8	High
27	Singh2013②^24^		7636	Prospective cohort study	Mayo Knee question	2, 5years	NOS‡‡	8	High
28	Valdes2012^30^		860	Cross-sectional study	WOMAC║	3.2 years	AHRQ††	6	Moderate
29	Sullivan2011^27^		120	Prospective cohort study	WOMAC║	12 months	NOS‡‡	8	High
30	Riddle2010^21^		140	Prospective cohort study	WOMAC║	6 months	NOS‡‡	8	High
31	Nilsdotter2009^15^		102	Prospective study	KOOS‡ SF-36**	6months, 12months, 5 years	AHRQ††	6	Moderate
32	Brander2003^2^		116	Prospective observational study	VAS* McGill Pain Questionnaire	1, 3, 6, 12 months	AHRQ††	11	High

Outcome Measurement Instrument: * VAS, visual analogue scale, † NRS, numerical rating scale, ‡ KOOS, knee injury and osteoarthritis outcome score., § OKS, oxford knee score, ║ WOMAC, western Ontario and McMaster osteoarthritis index, # KSS, knee society score, **SF-36, short form-36

Tools of Quality Evaluation: †† AHRQ, agency for healthcare research and quality, ‡‡ NOS, Newcastle Ottawa scale

### Methodologic quality of included reviews

The research primarily focused on high or medium-quality literature, with no low-quality literature included in the analysis. The quality of cohort studies was evaluated using the NOS scale, with ratings ranging from moderate (four) to high (nine). The case-control study received a score of six out of nine on the NOS scale, indicating a moderate level of evidence. Five cross-sectional studies were assessed for quality using AHRQ, with three receiving a high-quality rating and two receiving a moderate rating. The scores for these studies ranged from 6 to 11. In studies rated as moderate quality, the most frequent reasons were attributed to the presence of confounding and measurement bias. Nine cohort studies have not reported or controlled for confounders, which may have led to an elevated risk of confounding bias. Furthermore, four cross-sectional studies exhibited indications of measurement bias, and the handling of missing data were not disclosed in the publication. The quality evaluation of the included studies according to the NOS and AHRQ checklist are shown in Appendix 2.

### The level of evidence for risk factors

Twenty-nine risk factors associated with the incidence of postoperative chronic pain were identified. The results of the best evidence analysis are presented in Table 2. Upon conducting the study, it was found that ten variables exhibited a significant association with the onset of chronic pain following total knee arthroplasty (TKA). Age, body mass index (BMI), and comorbidities condition were discovered to possess strong evidence among demographic variables. As for preoperative factors, strong evidence was observed for preoperative pain, chronic widespread pain, preoperative adverse health beliefs, preoperative sleep disorders, central sensitization, preoperative anxiety, and preoperative function. No risk factors were strongly associated with the development of chronic pain among intraoperative and postoperative factors. Additionally, three factors were found to have a moderate association with outcome variables, namely gender, preoperative depression, and pain trajectory. At length, sixteen risk factors were identified as inconclusive, with the majority of them being statistically linked to chronic pain after TKA in just one study.

**Table 2:** Studies included in the systematic review

Risk factors	Multivariable analysis				Evidence grade
	High quality	Moderate quality	Low quality	No association	
**Patient characteristics**					
Age	^8, 26^	^15^			Strong
Gender	^3^	^30^			Moderate
BMI	^8, 10^	^29, 30^			Strong
Living in deprived areas	^10^				Inconclusive
History of previous diagnosed cancer	^26^				Inconclusive
Fibromyalgia	^26^				Inconclusive
History of previous knee surgery	^10^				Inconclusive
Comorbidities condition	^19 14, 25 10^				Strong
Years with knee pain		^30^			Inconclusive
**Perioperative Factors**					
**Preoperative Factors**					
Preoperative pain	^1, 2, 7, 11, 13, 17, 20, 26, 32^	^12^			Strong
Chronic widespread pain	^22, 26^	^6, 30^			Strong
Preoperative fatigue	^13^				Inconclusive
Preoperative adverse health beliefs	^13, 20, 21, 22, 27, 31, 32^	^12^			Strong
Preoperative sleep disorders	^16, 28^				Strong
Central sensitization	^16, 18^	^12^			Strong
Preoperative anxiety	^2, 3, 17, 25^				Strong
Preoperative depression	^25^	^30^			Moderate
Preoperative function	^3, 22, 26, 32^				Strong
Preoperative physical activity	^11^				Inconclusive
Ipsilateral lower extremity joint involvement	^24^				Inconclusive
Preoperative AT_2_R	^4^				Inconclusive
Preoperative opioid use	^13^				Inconclusive
Preoperative cortisol levels		^5^			Inconclusive
**Intraoperative Factors**					
ASA grade	^10^				Inconclusive
Duration of anesthesia	^13^				Inconclusive
**Postoperative Factors**					
Acute postoperative pain	^3^				Inconclusive
Epinephrine level		^5^			Inconclusive
Postoperative Coronal malalignment	^1^				Inconclusive
Pain trajectory	^23^	^9^			Moderate

BMI = Body mass index; ASA = American Society of Anesthesiologists grade; AT_2_R = angiotensin II type 2 receptor;

## Discussion

A total of 32 studies were included in our review, with a focus on case-control, cohort, and cross-sectional studies, and the grade of evidence in the literature was evaluated using the NOS scale, a quality assessment tool for cohort/case-control studies, and the AHRQ, a quality assessment tool for cross-sectional studies. Overall, the quality level of literature included in this study was high, and the reason for articles with a moderate level of evidence rating was the presence of potential confounding bias or measurement bias in the study. Twenty-nine risk factors connected with the development of chronic postoperative pain were identified, among which ten exhibited a strong correlation, three showed a moderate correlation, and sixteen factors yielded inconclusive results.

We employed a semi-quantitative approach to evaluate the level of evidence for risk factors and, in contrast to previous studies, identified two novel factors that exhibit a strong association with chronic pain following knee replacement surgery: preoperative sleep disturbances and preoperative poor health beliefs.

According to recent research that utilized machine learning and a large sample size, it has been determined that sleep problems can have a significant impact on chronic pain [13]. When we sleep, our body’s natural pain relief system is activated, and any disruptions to this system due to sleep deprivation or disturbances can negatively affect it [14]. A study was conducted to delve deeper into the relationship between sleep quality before total knee arthroplasty surgery and postoperative chronic pain syndrome (CPSP) [15]. The findings revealed that individuals who experienced sleep problems before the surgery were more likely to report higher pain scores three months after the procedure. This highlights the importance of addressing any pre-existing sleep issues before undergoing surgery to minimize the risk of postoperative chronic pain.

Health beliefs are thoughts, attitudes, or expectations that influence the experience of health and illness and related behaviors. Predictors such as illness perception, pain catastrophizing, preoperative expectations, and coping attitudes were grouped into the category of preoperative health beliefs in our article. Seven high-quality articles and one moderately quality article have demonstrated a statistically significant correlation between preoperative negative health beliefs and chronic postoperative pain [16–23]. Research has shown that patients who experience greater levels of preoperative pain catastrophizing are more likely to suffer from moderate to severe pain after surgery. A study conducted by Giusti et al. has revealed that behavioral outcomes can forecast pain and functional outcomes up to 12 months after surgery [24]. Additionally, the study suggests that these outcomes partially mediate the relationship between catastrophizing and subsequent pain and function. Furthermore, a cohort study has identified the existence of psychological risk factors that may hinder the implementation of proper pain coping strategies and lead to the development of chronic postoperative pain.

Our review identified sixteen factors with insufficient evidence, as they were only statistically associated with CPSP in one study upon critical appraisal and lacked support from other literature. This highlights the necessity for further validation of these under-evidenced factors in future studies, specifically investigating their association with chronic pain. Moreover, it is crucial to prioritize factors backed by robust evidence and develop interventional clinical protocols based on these high-risk factors to provide comprehensive guidance to clinicians and nurses.

### Limitations

This study has several limitations. In this systematic review, we only included patients with primary TKA and excluded those undergoing revision surgery and uni-compartmental arthroplasty; therefore, our findings may not extrapolate to other types of patients.

One of the major challenges in our study was the heterogeneity in the design of the included studies. We also found variations in the outcome indicators and measurement techniques used, which might account for the discrepancies in the results and hinder the integration of these findings.

Furthermore, we observed that some of the studies analyzed in this review did not adjust for potential confounders in their analyses. Confounding could have contributed to bias in our findings to some extent. Therefore, we recommend that future studies should put these factors into consideration when analyzing their results.

### Clinical implications

This systematic review can inspire future personalized pain prevention and management measures. Enhanced monitoring of patient-reported pain before and early after surgery may lead to early detection and potential early intervention of patients at risk for CPSP. Early identification and targeted treatment of pain may reduce pain and prevent long-term disability. Improving awareness of the importance of biological, sociocultural, psychological, physical, and clinical factors will help to implement the role of interventions better.

## Conclusion

This systematic review aims to assess the risk factors that contribute to the emergence of chronic pain after total knee arthroplasty. It further endeavors to appraise the evidence supporting these factors quantitatively. This analysis strives to enlighten surgeons and patients alike on potential risk factors that deserve exploration in future TKA pain management research, particularly those that have generated controversy or displayed weak correlations. Importantly, it underscores the necessity for additional high-quality studies to confirm these factors, thereby equipping clinicians with crucial knowledge regarding high-risk patients and their clinical characteristics. In turn, this knowledge contributes to the formulation of effective preventive measures aimed at reducing postoperative pain following TKA.

### Electronic supplementary material

Below is the link to the electronic supplementary material.

Supplementary Material 1

Supplementary Material 2

## Data Availability

No datasets were generated or analysed during the current study.
