# Supplementary material for: Risk factors of chronic postoperative pain after total knee arthroplasty: a systematic review
Source: J Orthop Surg Res. 2024 May 29;19:320. doi: 10.1186/s13018-024-04778-w (PMC11134678; doi:10.1186/s13018-024-04778-w)
Supplement: Supplementary file 1 — Supplementary Material 1 [file 13018_2024_4778_MOESM1_ESM.docx]

**Appendix 1: Search terms**

**Pubmed 2387 (** **1979** **to 31 July 2023 )**

#1 Search: Total knee arthroplasty or knee replacement or knee arthroplasty or joint replacement or knee surgery or joint surgery or Total Joint Arthroplasty or joint or total joint replacement or Joint Prosthesis Implantation* or Replacement Arthroplast* or Joint Replacement* or Total Joint Replacement* or Knee joint pain OR Arthroplasty, Replacement[Mesh]

#2 Search: **long-term pain or persistent pain or Postsurgical Pain or Post-operative Pain* or Postoperative Pain or Chronic Postoperative Pain or Chronic Post-surgical Pain or Chronic Postsurgical Pain* or Persistent Postsurgical Pain or Chronic Pain* or Widespread Chronic Pain* or Constant pain or Continual Pain OR Pain, Postoperative[Mesh]**

#3 Search: #1 AND #2

#4 Search**:** **predictive factors or risk factor or predictors or determinant or factor or predict* or associate* or assessment or cause or effect* or Social Risk Factor* or Health Correlates or Risk Assessment or predictive model OR Risk Factors[Mesh]**

#5 Search**:** #3 AND #4

#6 Search**: cross-sectional stud* OR cohort stud* OR case control stud***

#7 Search**:** #5 AND #6

**Web of science 11,231 (1927 to 31 July 2023)**

7

#6 AND #5 and Preprint Citation Index

11,231

6

TS=(cross-sectional stud* OR cohort stud* OR case control stud*) and Preprint Citation Index

3,072,602

5

#4 AND #3 and Preprint Citation Index 67,256

4

TS=（predictive factors or risk factor or predictors or determinant or factor or predict* or associate* or assessment or cause or effect* or Social Risk Factor* or Health Correlates or Risk Assessment or predictive model OR Risk Factors[Mesh]） and Preprint Citation Index

60,976,670

3

#1 AND #2 and Preprint Citation Index 86,603

2

TS=（long-term pain or persistent pain or Postsurgical Pain or Post-operative Pain* or Postoperative Pain or Chronic Postoperative Pain or Chronic Post-surgical Pain or Chronic Postsurgical Pain* or Persistent Postsurgical Pain or Chronic Pain* or Widespread Chronic Pain* or Constant pain or Continual Pain OR Pain, Postoperative[Mesh]） and Preprint Citation Index 481,918

1

TS=（Total knee arthroplasty or knee replacement or knee arthroplasty or joint replacement or knee surgery or joint surgery or Total Joint Arthroplasty or joint or total joint replacement or Joint Prosthesis Implantation* or Replacement Arthroplast* or Joint Replacement* or Total Joint Replacement* or Knee joint pain OR Arthroplasty, Replacement[Mesh]） and Preprint Citation Index 4,632,878

**CINAHL 2156 (1993 to 31 July 2023)**

TX ( cross-sectional stud* OR cohort stud* OR case control stud* ) AND AB ( Total knee arthroplasty or knee replacement or knee arthroplasty or joint replacement or knee surgery or joint surgery or Total Joint Arthroplasty or joint or total joint replacement or Joint Prosthesis Implantation* or Replacement Arthroplast* or Joint Replacement* or Total Joint Replacement* or Knee joint pain OR Arthroplasty, Replacement[Mesh] ) AND AB ( long-term pain or persistent pain or Postsurgical Pain or Post-operative Pain* or Postoperative Pain or Chronic Postoperative Pain or Chronic Post-surgical Pain or Chronic Postsurgical Pain* or Persistent Postsurgical Pain or Chronic Pain* or Widespread Chronic Pain* or Constant pain or Continual Pain OR Pain, Postoperative[Mesh] ) AND AB ( predictive factors or risk factor or predictors or determinant or factor or predict* or associate* or assessment or cause or effect* or Social Risk Factor* or Health Correlates or Risk Assessment or predictive model OR Risk Factors[Mesh] )

**EMBASE 1178 (1974 to 31 July 2023)**

1 (Total knee arthroplasty or knee replacement or knee arthroplasty or joint replacement or knee surgery or joint surgery or Total Joint Arthroplasty or joint or total joint replacement or Joint Prosthesis Implantation* or Replacement Arthroplast* or Joint Replacement* or Total Joint Replacement* or Knee joint pain).mp. [mp=title, abstract, heading word, drug trade name, original title, device manufacturer, drug manufacturer, device trade name, keyword heading word, floating subheading word, candidate term word] 626197

2 exp *replacement arthroplasty/ 27550

3 (long-term pain or persistent pain or Postsurgical Pain or Post-operative Pain* or Postoperative Pain or Chronic Postoperative Pain or Chronic Post-surgical Pain or Chronic Postsurgical Pain* or Persistent Postsurgical Pain or Chronic Pain* or Widespread Chronic Pain* or Constant pain or Continual Pain).mp. [mp=title, abstract, heading word, drug trade name, original title, device manufacturer, drug manufacturer, device trade name, keyword heading word, floating subheading word, candidate term word] 205608

4 exp *postoperative pain/su, th [Surgery, Therapy] 2074

5 1 or 2 633149

6 3 or 4 205608

7 (predictive factors or risk factor or predictors or determinant or factor or predict* or associate* or assessment or cause or effect* or Social Risk Factor* or Health Correlates or Risk Assessment or predictive model).mp. [mp=title, abstract, heading word, drug trade name, original title, device manufacturer, drug manufacturer, device trade name, keyword heading word, floating subheading word, candidate term word] 21089030

8 exp *risk factor/ 126191

9 7 or 8 21089030

10 5 and 6 and 9 14123

11 (cross-sectional stud* or cohort stud* or case control stud*).mp. [mp=title, abstract, heading word, drug trade name, original title, device manufacturer, drug manufacturer, device trade name, keyword heading word, floating subheading word, candidate term word] 1328334

12 10 and 11 1178

**Cochrane library 1840 (1993 to 31 July 2023)**

#1 MeSH descriptor: [Arthroplasty, Replacement] this term only

#2 (“Total knee arthroplasty” or “knee replacement” or “knee arthroplasty” or “joint replacement” or “knee surgery” or “joint surgery” or “Total Joint Arthroplasty” or “joint“ or “total joint replacement” or “Joint Prosthesis Implantation*” or “Replacement Arthroplast*” or “Joint Replacement*” or “Total Joint Replacement*” or “Knee joint pain“):ti,ab,kw

#3 #1 OR #2

#4 MeSH descriptor: [Pain, Postoperative] this term only

#5 (“long-term pain” or “persistent pain” or “Post-surgical Pain” or “Post surgical Pain” or “Postsurgical Pain” or “Post-operative Pain*” or “Post operative Pain” or “Postoperative Pain” or “Chronic Postoperative Pain” or “Chronic Post-surgical Pain” or “Chronic Post surgical Pain” or “Chronic Postsurgical Pain*” or “Persistent Postsurgical Pain” or “Chronic Post-operative Pain” or “Chronic Post operative Pain” or “Chronic Pain*” or “Widespread Chronic Pain*“ or “Constant pain” or “Continual Pain”):ti,ab,kw

#6 #4 OR #5

#7 #3 AND #6

#8 MeSH descriptor: [Risk Factors] this term only

#9 (“predictive factors” or “risk factor” or ”predictors” or “determinant” or “factor” or “predict*” or “associate*” or “assessment” or “cause” or “effect*” or “Social Risk Factor*” or “Health Correlates” or “Population* at Risk” or “Risk Assessment” OR “predictive model”):ti,ab,kw

#10 #8 OR #9

#11#7 AND #10

#12 (cross-sectional stud* OR cohort stud* OR case control stud*):ti,ab,kw

#13 #11 AND #12
