# Supplementary material for: Risk factors of chronic postoperative pain after total knee arthroplasty: a systematic review
Source: J Orthop Surg Res. 2024 May 29;19:320. doi: 10.1186/s13018-024-04778-w (PMC11134678; doi:10.1186/s13018-024-04778-w)
Supplement: Supplementary file 2 — Supplementary Material 2 [file 13018_2024_4778_MOESM2_ESM.docx]

**TABLE S1.** Results of quality assessment using the Newcastle Ottawa Scale for cohort studies

| Study | Selection | | | | Comparability | Outcome | | | Scores | Quality |
| --- | --- | --- | --- | --- | --- | --- | --- | --- | --- | --- |
|  | Demonstration that outcome of interest was not present at start of study | Representativeness of the exposed cohort | Selection of the non exposed cohort | Ascertainment of exposure | Comparability of cohorts on the basis of the design or analysis | Assessment of outcome | Was follow-up long enough for outcomes to occur | Adequacy of follow-up of cohorts |  |  |
| Yan2023 | ★ | ★ | ★ | ★ |  | ★ | ★ | ★ | 7 | high |
| Nishimoto2023 | ★ | ★ | ★ | ★ | ★★ | ★ | ★ | ★ | 9 | high |
| Chen2021 | ★ | ★ | ★ | ★ | ★★ | ★ | ★ | ★ | 9 | high |
| Larsen2021 | ★ | ★ |  | ★ |  | ★ | ★ | ★ | 6 | moderate |
| Aso2021 | ★ | ★ | ★ | ★ | ★★ | ★ | ★ | ★ | 9 | high |
| Imai2021 | ★ | ★ | ★ | ★ |  |  |  |  | 4 | moderate |
| Skrejborg2019 | ★ | ★ | ★ | ★ | ★★ | ★ | ★ | ★ | 9 | high |
| Singh2019 | ★ | ★ | ★ | ★ | ★★ | ★ | ★ | ★ | 9 | high |
| Gungor2019 | ★ | ★ | ★ | ★ |  | ★ |  |  | 5 | moderate |
| Buvanendran2019 | ★ | ★ | ★ | ★ | ★★ | ★ | ★ | ★ | 9 | high |
| Shim2018 | ★ |  |  | ★ | ★★ | ★ | ★ | ★ | 7 | high |
| Rice2018 | ★ | ★ | ★ | ★ | ★★ | ★ | ★ | ★ | 9 | high |
| Kornilov2018 | ★ | ★ | ★ | ★ | ★★ | ★ | ★ | ★ | 9 | high |
| Hofstede2018 | ★ |  |  | ★ | ★★ | ★ | ★ | ★ | 7 | high |
| Jiang2017 | ★ |  | ★ | ★ | ★★ | ★ | ★ | ★ | 8 | high |
| Dave2017 | ★ |  | ★ | ★ |  | ★ | ★ | ★ | 6 | moderate |
| Cremeans2016 | ★ |  | ★ | ★ |  | ★ | ★ | ★ | 6 | moderate |
| Rajamäki2015 | ★ |  | ★ | ★ |  | ★ | ★ | ★ | 6 | moderate |
| Petersen2015 | ★ | ★ | ★ | ★ |  | ★ | ★ | ★ | 7 | high |
| Nashi2014 | ★ | ★ | ★ | ★ | ★★ | ★ | ★ | ★ | 9 | high |
| Yakobo2014 | ★ |  | ★ | ★ | ★★ | ★ | ★ | ★ | 8 | high |
| Noiseux2014 | ★ |  | ★ | ★ | ★★ | ★ | ★ | ★ | 8 | high |
| Singh2013① | ★ |  | ★ | ★ | ★★ | ★ | ★ | ★ | 8 | high |
| Singh2013② | ★ |  | ★ | ★ | ★★ | ★ | ★ | ★ | 8 | high |
| Sulliva2011 | ★ |  | ★ | ★ | ★★ | ★ | ★ | ★ | 8 | high |
| Riddle2010 | ★ |  | ★ | ★ | ★★ | ★ | ★ | ★ | 8 | high |

**TABLE S2**. Results of quality assessment using the Newcastle Ottawa Scale for case-control studies

| Study | Selection | | | | Comparability | Exposure | | | Scores | Quality |
| --- | --- | --- | --- | --- | --- | --- | --- | --- | --- | --- |
|  | Is the case definition adequate? | Representativeness of the cases | Selection  of controls | Definition of controls | Comparability of cases and controls on the basis of the design or analysis | Ascertainment of exposure | Same method of ascertainment for cases and controls | Non-Response rate |  |  |
| Teimouri2023 | ★ | ★ | ★ | ★ |  | ★ | ★ |  | 6 | moderate |

**TABLE S3**. Results of quality assessment using the AHRQ(Agency for Healthcare Research and Quality) for cross-sectional studies

| Study | 1)Define the source of information (survey, record review) | 2)List inclusion and exclusion criteria for exposed and unexposed subjects (cases and controls) or refer to previous publications | 3)Indicate time period used for identifying patients | 4)Indicate whether or not subjects were consecutive if not population-based | 5)Indicate if evaluators of subjective components of study were masked to other aspects of the status of the participants | 6)Describe any assessments undertaken for quality assurance purposes (e.g., test/retest of primary outcome measurements) | 7)Explain any patient exclusions from analysis | 8)Describe how confounding was assessed and/or controlled. | 9) If applicable，explain how missing data were handled in the analysis | 10)Summarize patient response rates and completeness of data collection | 11)Clarify what follow-up, if any, was expected and the percentage of patients for which incomplete data or follow-up was obtained | Scores | Quality |
| --- | --- | --- | --- | --- | --- | --- | --- | --- | --- | --- | --- | --- | --- |
| Tang2023 | Yes | Yes | Yes | Yes | Yes | Unclear | Yes | No | No | Yes | Yes | 8 | high |
| Lindberg2021 | Yes | Yes | Yes | Yes | Unclear | Yes | Yes | No | No | Yes | Yes | 8 | high |
| Valdes2012 | Yes | Yes | Yes | Yes | Yes | Yes | No | No | No | No | No | 6 | moderate |
| Nilsdotter2009 | Yes | No | Yes | Yes | Unclear | No | Yes | No | No | Yes | Yes | 6 | moderate |
| Brander2003 | Yes | Yes | Yes | Yes | Yes | Yes | Yes | Yes | Yes | Yes | Yes | 11 | high |
